# Hepatoma cell-intrinsic TLR9 activation induces immune escape through PD-L1 upregulation in hepatocellular carcinoma

**DOI:** 10.7150/thno.44417

**Published:** 2020-05-17

**Authors:** Binghai Zhou, Jiuliang Yan, Lei Guo, Bo Zhang, Shuang Liu, Mincheng Yu, Zheng Chen, Kewei Zhang, Wentao Zhang, Xiaoqiang Li, Yongfeng Xu, Yongsheng Xiao, Jian Zhou, Jia Fan, Mien-Chie Hung, Hui Li, Qinghai Ye

**Affiliations:** 1 Department of Liver Surgery and Transplantation, Liver Cancer Institute, Zhongshan Hospital, Fudan University, Key Laboratory of Carcinogenesis and Cancer Invasion (Fudan University), Ministry of Education, Shanghai, China.; 2 Department of Neurosurgery, Zhongshan Hospital, Fudan University, Shanghai, China.; 3 Department of General Surgery, Xinhua Hospital, School of Medicine, Shanghai Jiao Tong University, Shanghai, China.; 4 Department of Thoracic Surgery, Peking University Shenzhen Hospital, Shenzhen, China.; 5 Graduate Institute of Biomedical Sciences and Center for Molecular Medicine, China Medical University, Taichung 404, Taiwan.

**Keywords:** PD-L1, Hepatocellular carcinoma, TLR9, PARP1, STAT3

## Abstract

A TLR9 agonist in combination with a PD-1 inhibitor produced powerful antitumor responses in a clinical trial despite TLR9 agonists as monotherapies failing to generate systemic antitumor immune responses due to immunosuppressive effects. However, the mechanism involved in the improved response induced by their combination remains unknown.

**Methods**: Subcutaneous and orthotopic Hepa1-6 tumor model was used for single-drug and combined-drug treatment. We used TLR9 agonist stimulation or lentiviral vectors to overexpress TLR9 and activate TLR9 signaling. We next investigated the crosstalk between PARP1 autoPARylation and ubiquitination and between STAT3 PARylation and phosphorylation mediated by TLR9. Tissue chips were used to analyze the relationships among TLR9, PARP1, p-STAT3 and PD-L1 expression.

**Results**: In this study, we found that the TLR9 agonist in combination with anti-PD-1 therapy or anti-PD-L1 therapy yielded an additive effect that inhibited HCC growth in mice. Mechanistically, we found that TLR9 promoted PD-L1 transcription by enhancing STAT3 Tyr705 phosphorylation. Then, we observed that TLR9 negatively regulated PARP1 expression, which mediated a decrease in STAT3 PARylation and an increase in STAT3 Tyr705 phosphorylation. Moreover, we found that TLR9 enhanced PARP1 autoPARylation by inhibiting PARG expression, which then promoted the RNF146-mediated ubiquitination and subsequent degradation of PARP1. Finally, we observed positive associations between TLR9 and p-STAT3 (Tyr705) or PD-L1 expression and negative associations between TLR9 and PARP1 in HCC patient samples.

**Conclusions**: We showed that hepatoma cell-intrinsic TLR9 activation regulated the crosstalk between PARP1 autoPARylation and ubiquitination and between STAT3 PARylation and phosphorylation, which together upregulated PD-L1 expression and finally induces immune escape. Therefore, combination therapy with a TLR9 agonist and an anti-PD-1 antibody or anti-PD-L1 had much better antitumor efficacy than either monotherapy in HCC.

## Introduction

Hepatocellular carcinoma (HCC) is the fifth most common cancer in the world and the second leading cause of cancer-related death 1. Currently, treatments for early-stage HCC include surgical resection, liver transplantation and local radiofrequency (RF) ablation 2, but their efficacy remains limited. Molecular targeted therapies such as the small-molecule multikinase inhibitor sorafenib (first-line use) 3, regorafenib (second-line use) 4 and lenvatinib (first-line use) 5 have been approved by the US Food and Drug Administration (FDA) for the treatment of advanced HCC. However, these drugs only extend the median overall survival of advanced HCC patients by no longer than 4 months, and the overall response rate is extremely low 6. Immune checkpoint blockade (ICB) has yielded considerable clinical benefits in patients with different tumors by enhancing T cell responses and maintaining prolonged antitumor activity 7-10. The anti-PD1 therapy approved for HCC treatment has achieved a 20% response rate 11. However, treatment with pembrolizumab or nivolumab failed to meet the primary endpoints of the KEYNOTE-240 and CheckMate-459 HCC clinical trials. Therefore, there is an urgent need to improve the therapeutic effect of ICB and develop more effective combination therapies.

Toll-like receptors (TLRs) are important sensors of pathogen-associated molecular patterns that help to protect the host from foreign intruders. Engagement of TLRs links innate immunity with adaptive immunity and results in protective immunity induction 12. This principle has subsequently been used and exploited therapeutically, thus making TLR agonists important components of current cancer immunotherapy 13. For example, triggering TLR responses (such as by administering a TLR9 agonist) in tumor cells can lead to the induction of tumor cells death 14, which, under certain conditions, is associated with antitumor immunity induction by mechanisms involving immunogenic or immunostimulatory cell death 15. However, numerous clinical trials using TLR9 agonists to generate systemic antitumor immune responses have generally been unsuccessful 16, 17. In the context of failed therapeutic applications, the expression of TLR9 on tumor cells and the consequences of TLR activation on cancer cells have received increasing attention. The induction of TLRs, especially TLR9, in tumor cells by endogenous ligands does not result in antitumor immunity but rather contributes to tumor progression 18-27. A recent study suggested that endosomal TLRs in tumor cells limited endogenous adaptive and protective T cell responses in the tumor-bearing host 13. Therefore, understanding the effects of TLR9 in tumor cells on antitumor immunity may lead to the discovery of new therapeutic targets for cancer therapy. Moreover, recent studies have found that TLR9 agonists can warm “cold” melanoma tumors and reverse ICB resistance, as these agonists can induce high levels of IFNα, which is associated with the transcriptional signature of tumors responsive to ICB 28, 29. Intratumoral injection of SD-101 (a TLR9 agonist) in combination with pembrolizumab produces powerful antitumor responses in patients with stage IIIC/IV melanoma 30. However, the specific mechanism involved in the improved response to TLR9 agonist and ICB combination treatment is not yet well understood.

Previous studies have reported that PD-L1 expression upregulation in tumor cells mediates immune tolerance and reduces tumor-infiltrating T cell killing ability, which may be responsible for resistance to molecular targeted drugs 31-33. In our previous study, we found that combining a MET inhibitor that upregulated PD-L1 expression with an anti-PD-1 antibody could yield an additive effect in an HCC mouse model 32. PD-L1 expression levels within the tumor microenvironment can predict treatment responses to monotherapies blocking the PD-L1/PD-1 axis in different tumor types 34-36, which are regulated in highly complex manners and can be influenced by transcriptional control and posttranslational regulation 37. STAT3, which can act directly on the PD-L1 promoter to increase PD-L1 expression in human cancer cells, has been shown to be one of the most important transcription factors involved 38. In addition, STAT3 activity has been reported to be negatively regulated by PARP1, which PARylates STAT3 and enhances STAT3 dephosphorylation, ultimately attenuating PD-L1 expression 39. High TLR9 expression and STAT3 activation are observed in polymorphonuclear myeloid-derived suppressor cells (PMN-MDSCs), which accumulate in the circulation to promote prostate cancer progression 40. Moreover, cell death causes the release of TLR9 ligands, such as mitochondrial DNA, and TLR9/NF-kB-induced secretion of IL6-type cytokines, which in turn stimulate STAT3 activity in cancer cells and myeloid cells in the tumor microenvironment to initiate cancer recurrence 19, 26. These findings indicate that TLR9 signaling leads to tumor progression, which may be highly related to STAT3 activation in the tumor microenvironment, but the mechanism by which TLR9 signaling activates STAT3 in cancer cells to inhibit antitumor immunity is poorly understood.

Herein, we found that anti-PD-1 therapy in combination with a TLR9 agonist improved antitumor activity in an HCC mouse model, which was in agreement with previous clinical trial results. Specifically, TLR9 promoted PD-L1 transcriptional expression by enhancing STAT3 Tyr705 phosphorylation, resulting in tumor cell immune escape. Moreover, we found crosstalk between STAT3 PARylation and phosphorylation, which meant that inhibiting PARP1 led to a decrease in STAT3 PARylation and an increase in STAT3 Tyr705 phosphorylation. Furthermore, we identified that TLR9 was a negative regulator of PARP1 that promoted RNF146-mediated PARP1 ubiquitination by inhibiting poly (ADP-ribose) glycohydrolase (PARG) to promote PARP1 autoPARylation. Our results reveal a novel mechanism in which TLR9 regulates the crosstalk between PARP1 autoPARylation and ubiquitination and between STAT3 PARylation and phosphorylation, which together mediate in PD-L1 expression and affect antitumor immunity.

## Materials and Methods

### Cell culture

The HCC cell lines Hep3B, Huh7 and Hepa1-6 were obtained from the Liver Cancer Institute, Fudan University, Shanghai, China. The cells were cultured in DMEM (Thermo Fisher Scientific, Waltham, MA, USA) supplemented with 10% fetal bovine serum (FBS; Gibco) and penicillin-streptomycin at 37 °C and in a humidified atmosphere with 5% CO2.

### Cell transfection

Small interfering RNAs (siRNAs) targeting human Myc, JUN, IRF1, IRF3, STAT1, STAT3 and RNF146 were synthesized by Genomeditech (Shanghai, China). PGMLV-CMV-H_PARG-3×Flag-EF1-ZsGreen1-T2A-Puro overexpression plasmid vector was constructed by Genomeditech (Shanghai, China). CMV-PARP1-EGFP-SV40-Neomycin overexpression plasmid vector and Ubi-H_TLR9-3FLAG-SV40-EGFP overexpression and hU6-TLR9-Ubiquitin-EGFP-IRES-puromycin knockdown lentiviral vectors was constructed by GeneChem Co., Ltd (Shanghai, China).

### Antibody and reagents

The antibodies listed below were used in Western blotting, immunohistochemical and flow cytometry analyses: anti-TLR9 (ab37154, Abcam, Cambridge, UK; NBP2-24729, Novus Biologicals, Minneapolis, USA), anti-PARP1 (#9532; Cell Signaling Technology, Danvers, MA, USA; ab227244, Abcam), anti-PAR (#83732; Cell Signaling Technology, Danvers, MA, USA), anti-Ubiquitin (#3936; Cell Signaling Technology, Danvers, MA, USA); anti-STAT3 (#9139; Cell Signaling Technology, Danvers, MA, USA), anti-p-STAT3 (Tyr705) (#9145; Cell Signaling Technology, Danvers, MA, USA); anti-PD-L1 (#13684T, Cell Signaling Technology, Danvers, MA, USA; 329702, BioLegend, San Diego, CA, USA; ab205921, Abcam, Cambridge, UK), anti-Jak2 (#3230; Cell Signaling Technology, Danvers, MA, USA), anti-p-Jak2 (#3774; Cell Signaling Technology, Danvers, MA, USA), anti-STAT1(#14994, Cell Signaling Technology, Danvers, MA, USA), anti-JUN(#9165; Cell Signaling Technology, Danvers, MA, USA), anti-IRF1(#8478; Cell Signaling Technology, Danvers, MA, USA), anti-IRF3(#11904; Cell Signaling Technology, Danvers, MA, USA); anti-Myc (Sigma-Aldrich, St. Louis, MO, USA), anti-granzyme B (ab4059; Abcam), anti-CD8 (ab22378; Abcam; 560776; BD Biosciences), anti-JNK (AF6318; Affinity Biosciences, USA), anti-p-JNK (AF3318; Affinity Biosciences, USA), anti-ERK (AF0155; Affinity Biosciences, USA), anti-p-ERK (AF1015; Affinity Biosciences, USA), anti-p-p38 (AF4001; Affinity Biosciences, USA), anti-p38 (AF6456; Affinity Biosciences, USA), anti-PARG (#27808; Proteintech, Wuhan), anti-RNF146 (#ARP43340; Aviva Systems Biology Co., Ltd. USA; #bs-11669R; Bioss Antibodies Inc. USA), anti-CD4 (550954; BD Biosciences), anti-PD-1 (551892; BD Biosciences), anti-CD11b (557395; BD Biosciences), anti-NK1.1 (557391; BD Biosciences), anti-CD25 (553071; BD Biosciences), anti-Foxp3 (560402; BD Biosciences), anti-F4/80 (565612;BD Bioscience), anti-CD11c (553800; BD Biosciences), anti-CD317 (566431; BD Biosciences), PDD-00017273 (#5952; Tocris Bioscience, USA), ODN2216 (#tlrl-2216; Invivogen, USA), ODN2243(ODN2216 Control, #tlrl-2243; Invivogen, USA) and SP600125 (#T3109), U0126(#T6223), SB203580(#T1764), BP-1-102 (#T3708), Chloroquine diphosphate (#T0194) were purchased from Topscience (Topscience Co., Ltd. Shanghai). PNGase F (#P0704) was purchased from New England Biolabs (New England Biolabs, Ipswich, MA, USA).

### Immunoprecipitation (IP) and Western blot analysis

IP and Western blot analysis were performed as described previously 32. In brief, for IP, liver cancer cells were lysed in a buffer (50mM Tris-HCl, pH 8.0; 150mM NaCl; 5mM ethylenediaminetetraacetic acid; and 0.5% Nonidet P-40). After removing cell debris, the indicated antibodies were added to clear the lysates with 25 μl of protein A/G agarose beads (#3159558; EMD Millipore Corp., USA). The samples were incubated on a rotating wheel overnight at 4 °C. The washed beads were boiled in a 5×SDS-polyacrylamide gel electrophoresis sample buffer. For Western blot analysis, band intensity quantitation for Western blotting was performed using ImageJ (National Institutes of Health, Bethesda, MD, USA). PVDF membranes were blocked with 5% milk, incubated with primary antibodies for 12-24h at 4 °C and then incubated with HRP-conjugated anti-mouse/rabbit secondary antibodies for 2 h after 3 washes with TBST. Low-abundance proteins were visualized with an enhanced chemiluminescence detection reagent (Thermo Fisher Scientific, Waltham, MA, USA).

### Real-time PCR assay

Total RNA was isolated by using Trizol reagent (Invitrogen, Carlsbad, CA, USA). Equal amounts of RNA were reverse transcribed into cDNA and amplified by PCR according to the manufacturer's protocol (Takara). qRT-PCR was performed using SYBR-Green PCR Master mix (Yeasen Biotechnology Co., Ltd.) according to the manufacturer's protocol. The primers used were as follows: human PD-L1 forward, 5'-GCTGCACTAATTGTCTATTGGGA-3' and reverse, 5'-AATTCGCTTGTAGTCGGCACC-3'; human GAPDH forward, 5ʹ-TGACTTCAACAGCGACACCCA-3ʹ and reverse, 5ʹ-CACCCTGTTGCTGTAGCCAAA-3ʹ; human PARP1 forward, 5'-AAGGCGAATGCCAGCGTTAC-3' and reverse, 5'-GGCACTCTTGGAGACCATGTCA-3'; Human TLR9 forward, 5'-CTGCCTTCCTACCCTGTGAG-3' and reverse, 5'-GGATGCGGTTGGAGGACAA-3'.

### Immunofluorescence

Huh 7 cells with wild-type or TLR9 overexpression were grown on coverslips in 12-well plates. When cells grown up to 90%, they were fixed in 4% paraformaldehyde for 20 min at room temperature, and permeabilized accompanying with blockage using 0.3% Triton X-100 with 10% bovine serum albumin for one hour. After blockage, cells were incubated with indicated antibodies at 4℃ over-night, and subsequently incubated with goat anti-mouse or anti-rabbit secondary antibody for one hour at room temperature. The coverslips were mounted with antifade reagent with DAPI and observed under a microscope.

### Immunohistochemistry

Tissue microarrays containing tumor and matched nontumor liver tissue samples were constructed as described previously 32. Briefly, tumor specimens were collected from HCC patients who underwent surgical resection from August 2001 to November 2007 in Liver Surgery Department of Zhongshan Hospital, Fudan University, Shanghai, China. All patients signed the informed consents and the protocols were approved by the Research Ethics Committee of Zhongshan Hospital. The study methodologies conformed to the standards set by the Declaration of Helsinki. Paraffin-embedded implanted tumors were cut into 5μm sections. Immunohistochemical (IHC) staining of the samples was performed as described previously. Tissue sections were incubated in 1x glycoprotein denaturing buffer for 3 hours at room temperature before addition of PD-L1 primary antibody, then washed 4 times with PBS, and treated with PNGase F (5%) containing PBS at 37 °C overnight. The purpose of this procedure is to remove the N-glycosylation modification on PD-L1 which has been reported enhancing PD-L1 detection after deglycosylation and predicting the therapeutic response of PD-1/PD-L1 41. In brief, each tissue sample was stained with specific antibodies as indicated and then incubated with an avidin-biotin-peroxidase complex. Visualization of the target protein was performed using the chromogen 3-amino-9-ethylcarbazole. The H-score method was used to score the samples by combining the values for immunoreaction intensity and percentage of tumor cell staining. The hybrid score formula was as follows: (% cells of 1 + intensity score × 1) + (% cells of 2 + intensity score × 2) + (% cells of 3 + intensity score × 3). The following four groups were created according to the histological scores: high (+++), medium (++), low (+), and negative (-).

### In vivo tumor experiments

Mouse Hepa1-6 liver cancer cells were injected (10^7^ cells transplanted subcutaneously (s.c.)) to grow tumors in C57BL/6 mice (male, 5-6 weeks old, weighing 20-22g). For the orthotopic tumor model, subcutaneous Hep1-6 tumors were cut into cubes (1mm^3^) under aseptic conditions. Single cubes were then inoculated into the liver parenchyma of C57BL/6 mice anesthetized using xylazine. This study was approved by the Shanghai Medical Experimental Animal Care Committee and performed according to the National Institutes of Health “Guide for the Care and Use of Laboratory Animals”. In order to mimic the antitumor effect of TLR9 agonist combined with immunotherapy accurately, we searched Clinical Trials.gov and found that a Class A TLR9 agonist (CMP-001) is used to combine with anti-PD-1 or anti-PD-L1 monoclonal antibody in melanoma (**NCT02680184**), colorectal cancer (**NCT03507699**) and lymphoma (**NCT03618641**). Therefore, we selected the murine (ODN1585) and the human (ODN2216) Class A TLR9 agonists for *vivo* and *vitro* experiments. The mice were randomly divided into groups, each containing 6 mice, after the tumors grew to 108-171.5 mm^3^ on average and were treated as follows: for antibody-based drug intervention, 100μg of anti-PD-1 antibody (RMP1-14; Bio X Cell, West Lebanon, NH, USA) or 100μg of anti-PD-L1 antibody (10F.9G2; Bio X Cell, West Lebanon, NH, USA) or rat IgG (control; Bio X Cell) was injected intraperitoneally every 3 days. For drug-based intervention, mice were given 30μg of TLR9 agonist ODN1585 (#tlrl-1585; Invivogen, USA) and ODN1585 Control (#tlrl-1585c; Invivogen, USA) were injected intraperitoneally every 3 days. Subcutaneous tumors were measured using a caliper twice a week. Tumor volumes were calculated using the formula: tumor volume = length × width^2^/2. At the end of the experiment, the mice were euthanized by cervical dislocation, and the tumors were obtained for subsequent histological and flow cytometric analyses.

### Statistics

Results are expressed as mean ± SD and all statistical tests were performed as 2 sided. For data normally distributed, we performed Student's t test, and the nonparametric exact Wilcoxon's signed-rank test was used to compare data not normally distributed. Cumulative survival time was estimated by the Kaplan-Meier method, and the log-rank test was applied to compare the groups. P < 0.05 was considered statistically significant. No animal data were excluded.

## Results

### Anti-PD-1 therapy in combination with a TLR9 agonist improved antitumor activity

**Figure 1:**
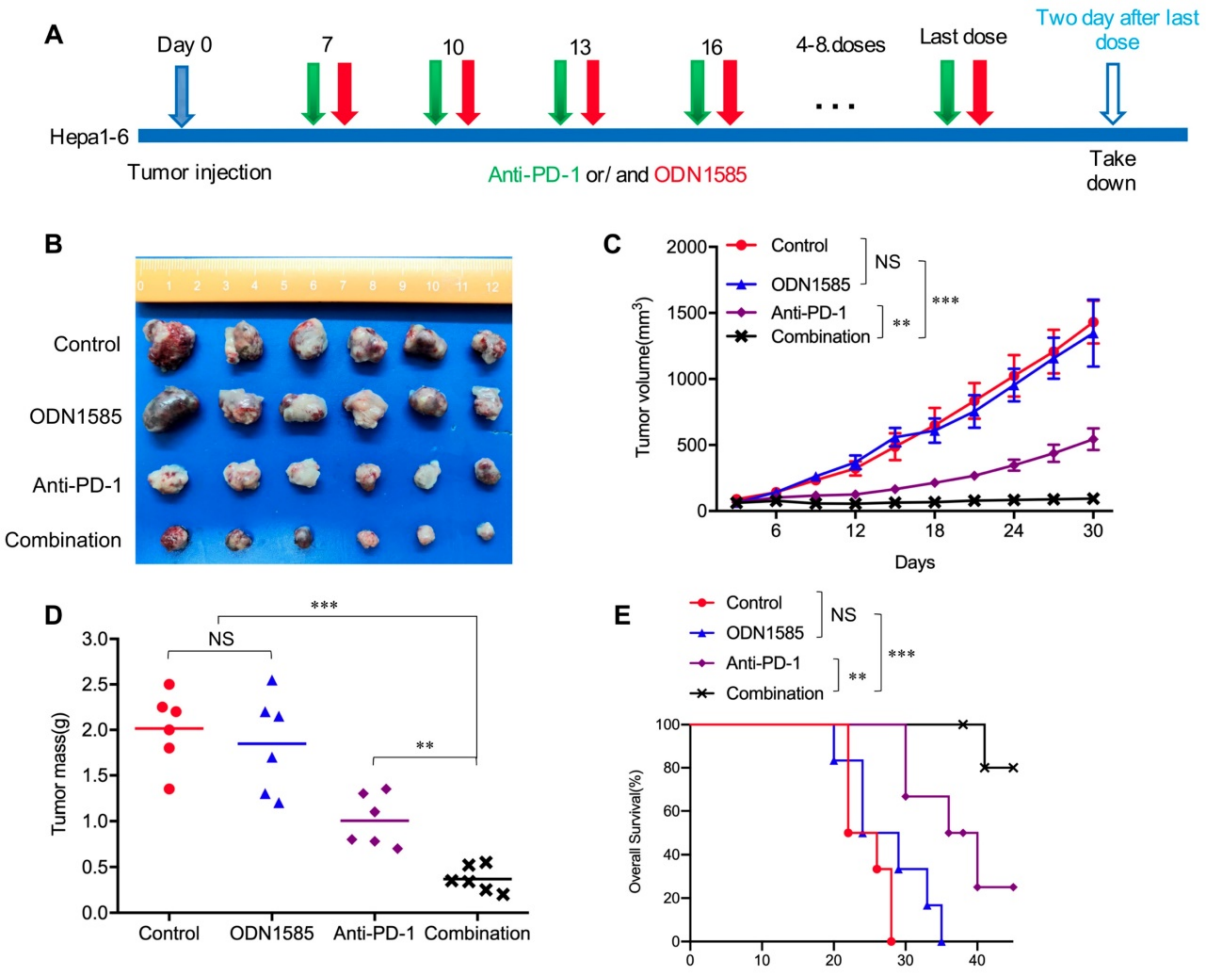
** Anti-PD-1 therapy in combination with a TLR9 agonist improved antitumor activity.** (**A**) Schematic diagram of the drug intervention protocol utilizing the TLR9 agonist ODN1585 and/or anti-PD-1 antibody to treat C57BL/6 mice. (**B**) Representative images of Hepa1-6 subcutaneous HCC tumors from each group (*n*=6 per group). (**C**) Growth of subcutaneous Hepa1-6 tumors in ODN1585- and/or anti-PD-1 antibody-treated C57BL/6 mice. Tumors were measured at the indicated time points. (*n*=6 per group, values are mean ± SD, **p < 0.01, ***p<0.001, NS indicates no significance). (**D**) Tumor weights at the drug intervention endpoints (*n*=6 per group, values are mean ± SD, **p < 0.01, ***p<0.001, NS indicates no significance). (**E**) Survival of mice bearing Hepa1-6 tumors following treatment with ODN1585 and/or anti-PD-1 antibody (*n*=6 per group, Significance was evaluated using the log-rank test. **p < 0.01, ***p<0.001, NS indicates no significance).

Recent studies have revealed that TLR9 agonists can warm “cold” melanoma tumors and reverse ICB resistance by expanding functional T cells, even though TLR9 agonists have been reported to induce immunosuppression 28-30. To determine whether anti-PD-1 therapy in combination with a TLR9 agonist enhances antitumor activity in an HCC mouse model, Subcutaneous and orthotopic Hepa1-6 tumor model was used for single-drug and combined-drug treatment. Before we conduct the combination therapy, we explored the dosage of anti-PD-1 monoclonal antibody in HCC mice model with 50μg, 100μg and 150μg doses respectively treated with TLR9 agonist. We found that there was no difference in antitumor effect between the 100μg dose and the 150μg doses group, but the tumors in 100μg group were significantly smaller than these in 50μg group. The results showed that 100μg doses is enough to block all the PD-1/PD-L1 binding even PD-L1 was increased after TLR9 agonist treatment whereas 50μg doses is not sufficient. Therefore, the dosage of 100 μg was determined in combination therapy (Figure S1A). We first treated mice bearing subcutaneous Hepa1-6 tumors with ODN1585 (a murine TLR9 agonist) or an anti-PD-1 antibody alone or in combination and monitored tumor growth (Figure 1A). ODN1585 failed to significantly reduce the tumor burden, and the anti-PD-1 antibody slightly restricted tumor growth, but the combination treatment showed much better antitumor efficacy than control treatment or the monotherapies (Figure 1B-D). In addition, compared with each treatment alone, treatment with both ODN1585 and the anti-PD-1 antibody substantially prolonged the overall survival of mice bearing subcutaneous Hepa1-6 tumors (Figure 1E).

To further validate our findings in vivo and vitro, we first performed experiments to find out whether TLR9 agonist alone or combination with anti-PD-L1 influence proliferation or apoptosis of HCC cells in vitro. We found that TLR9 agonists, anti-PD-L1 and combination drugs failed to inhibit the proliferation and apoptosis of HCC cells (Figure S1B-D). We then administered ODN1585 or anti-PD-1/anti-PD-L1 antibody or in combination to mice bearing orthotopic Hepa1-6 tumors and analyzed the tumor size (Figure S1E). We found that both of the combination of anti-PD-1 or anti-PD-L1 and ODN1585 impaired tumor growth and reduced tumor burden more effectively than in control mice or mice receiving ODN1585 or anti-PD-1 or anti-PD-L1 antibody alone (Figure S1F-H). Similarly, ODN1585 was also observed failed to significantly reduce the tumor burden in mice bearing the orthotopic Hepa1-6 tumors (Figure S1F-H). Moreover, the combination of TLR9 agonist and anti-PD-1 or anti-PD-L1 prolonged the overall survival of mice bearing the orthotopic Hepa1-6 tumors compared with ODN1585 or anti-PD-1 or anti-PD-L1 antibody alone (Figure S1I). These results illustrated that the ODN1585 and anti-PD-1 antibody or anti-PD-L1 antibody combination indeed improved antitumor activity in the HCC mouse subcutaneous and orthotopic tumor model, which was consistent with animal research and clinical trial results 29, 30, 42.

### TLR9 activation upregulated PD-L1 expression by promoting STAT3 Tyr705 phosphorylation in HCC cells

**Figure 2:**
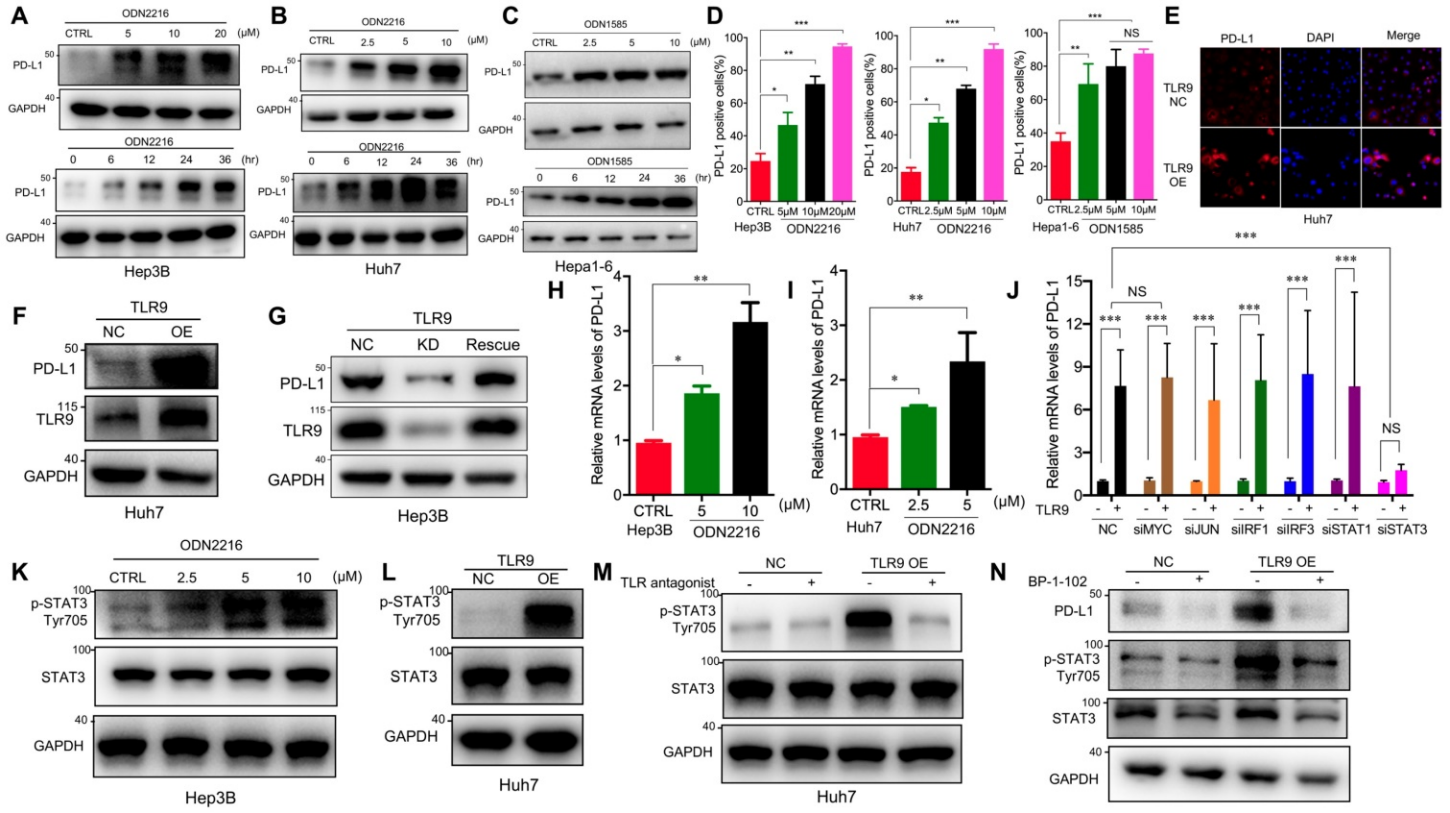
** TLR9 activation upregulated PD-L1 expression by promoting STAT3 Tyr705 phosphorylation in HCC cells.** (**A**) PD-L1 protein expression after treatment with ODN2216 with different concentrations or the indicated times (different concentrations: 0, 5, 10, or 20μM; times: 0, 6, 12, 24, and 36 hours in 10 μM) in Hep3B cells. PD-L1 protein levels were analyzed by Western blotting. (**B**) PD-L1 protein expression after treatment with ODN2216 with different concentrations or the indicated times (different concentrations: 0, 2.5, 5, or 10μM; times: 0, 6, 12, 24, and 36 hours in 10 μM) in Huh7 cells. PD-L1 protein levels were analyzed by Western blotting. (**C**) PD-L1 protein expression after treatment with ODN1585 with different concentrations or the indicated times (different concentrations: 0, 2.5, 5, and 10 μM; times: 0, 6, 12, 24, and 36 hours in 5μM) in Hepa1-6 cells. PD-L1 protein levels were analyzed by Western blotting. (**D**) PD-L1^+^ tumor cells were detected by flow cytometry after TLR9 agonist (Hep3B and Huh7 cells with ODN2216; Hepa1-6 cells with ODN1585) treatment with indicated concentration. (values are mean ± SD, *p < 0.05, **p < 0.01, ***p<0.001, NS indicates no significance). (**E**) PD-L1 expression after TLR9 overexpression in Huh7 cells. PD-L1 expression levels were analyzed by immunofluorescence. (**F**) PD-L1 protein expression after TLR9 overexpression in Huh7 cells. PD-L1 protein levels were analyzed by Western blotting. (**G**) PD-L1 protein expression in TLR9 knockdown or TLR9 rescue Hep3B cells. PD-L1 protein levels were analyzed by Western blotting. (**H** and **I**) mRNA levels of PD-L1 in Hep3B (**H**) and Huh7 (**I**) cells measured by qRT-PCR after stimulation with different concentrations of ODN2216. (values are mean ± SD, *p < 0.05, **p < 0.01, NS indicates no significance) (**J**) TLR9 overexpression-induced PD-L1 expression after MYC, JUN, IRF1, IRF3, STAT1 or STAT3 silencing. PD-L1 mRNA expression in Huh7 cells was analyzed after TLR9 overexpression alone or in the presence of MYC-, JUN-, IRF1-, IRF3-, STAT1- or STAT3-specific siRNA or siRNA-NC. (values are mean ± SD, ***p<0.001). (K) p-STAT3 (Tyr705) levels in Hep3B cells after treatment with different concentrations of ODN2216 (a TLR9 agonist; ODN2216: 0, 2.5, 5, or 10μM) analyzed by Western blotting. (**L**) p-STAT3 (Tyr705) levels after TLR9 overexpression in Huh7 cells analyzed by Western blotting. (**M**) TLR9 overexpression-induced p-STAT3 (Tyr705) levels after TLR9 inhibition. p-STAT3 (Tyr705) levels were analyzed after TLR9 overexpression alone or in the presence of the TLR9 antagonist chloroquine diphosphate. (**N**) p-STAT3-induced PD-L1 levels after STAT3 inhibition. PD-L1 levels were analyzed after TLR9 overexpression alone or in the presence of the STAT3-specific small molecular inhibitor BP-1-102.

Previous studies have shown that PD-L1 expression upregulation in tumor cells mediates immune tolerance and inhibits the killing ability of tumor-infiltrating T cells, which may be one of the reasons for tumor cell resistance to molecular targeted drugs 32, 33. To determine whether TLR9 activation regulates PD-L1 expression, we directly detected PD-L1 expression in HCC cells after TLR9 agonist stimulation. We observed that PD-L1 protein levels were significantly increased in a dose- and time-dependent manner in HCC cells following ODN2216 (a human TLR9 agonist, stimulating Hep3B, Huh7 cells) or ODN1585 (a murine TLR9 agonist, stimulating Hepa1-6 cells) treatment (Figure 2A-C). Meanwhile, we observed that the proportion of PD-L1 positive liver cancer cells (Hep3B, Huh7 and Hepa1-6 cells) increased significantly (Figure 2D). Since HCC patients with high PD-L1 expression (>5% of HCC cells) had significantly shorter overall survival time than that patients with low PD-L1 expression (<5% of HCC cells), we focused on the relationship between TLR9 and PD-L1 in HCC for all subsequent experiments. We first found that patients with low TLR9 and low PD-L1 expression pattern had the longest OS, whereas patients with the high TLR9 and high PD-L1 expression pattern had the shortest OS (Figure S2A).

Then, we detected PD-L1 expression by Western blotting and immunofluorescence after exogenously overexpressing TLR9 (Figure S2B) in Huh7 cells, and the results showed that PD-L1 expression was significantly increased after TLR9 overexpression (Figure 2E-F). We further established stable TLR9 knockdown Hep3B cell line with lentiviral vectors and found that PD-L1 expression was significantly reduced when loss of TLR9. In addition, we rescue TLR9 expression in TLR9 knockdown Hep3B cells and found that PD-L1 expression was significantly increased after TLR9 rescue (Figure 2G). Moreover, PD-L1 mRNA expression was quantified by real-time PCR after TLR9 agonist stimulation or TLR9 overexpression. The results showed that PD-L1 mRNA expression was increased after TLR9 activation in Hep3B and Huh7 cells (Figure 2H-I and Figure S2C-D). We then analyzed the correlation between TLR9 and PD-L1 by using the Signature Score Function at the GEPIA server 43. The result showed that a positive correlation of TLR9 expression and PD-L1 mRNA expression (*p*=0.00086, Figure S2E), implying that the regulation between TLR9 and PD-L1 may occur at the transcriptional level. Together, these results indicate that TLR9 activation enhances PD-L1 transcription in HCC cells.

A number of transcription factors, including MYC, JUN, IRF1, IRF3, STAT1 and STAT3, have been shown to be involved in regulating PD-L1 transcription by directly binding to its promoter region. To understand the molecular mechanisms by which TLR9 enhances PD-L1 transcription in HCC, we silenced the transcription factors MYC, JUN, IRF1, IRF3, STAT1 and STAT3 by gene inhibition. We found that PD-L1 expression induced by TLR9 activation was not affected by MYC, JUN, IRF1, IRF3, or STAT1 deficiency but was abolished by STAT3 silencing (Figure 2J and Figure S2F-K). Then, we found that STAT3 Tyr705 phosphorylation was significantly upregulated after ODN2216 stimulation in Hep3B cells (Figure 2K). Furthermore, we performed Western blotting and immunofluorescence and found that STAT3 Tyr705 phosphorylation was significantly upregulated in TLR9-overexpressing Huh7 cells (Figure 2L and Figure S2L). Furthermore, we blocked TLR9 with a TLR9 antagonist and found a significant decrease in STAT3 Tyr705 phosphorylation (Figure 2M), suggesting that TLR9 activation promotes STAT3 Tyr705 phosphorylation in HCC cells. To investigate whether the TLR9-mediated upregulation of PD-L1 expression is dependent on STAT3 Tyr705 phosphorylation in HCC, we further inhibited STAT3 with selective small-molecule inhibitors in TLR9-overexpressing Huh7 cells. PD-L1 expression was decreased when STAT3 Tyr705 phosphorylation was inhibited (Figure 2N). In summary, these results suggest that the upregulation of PD-L1 expression induced by TLR9 activation is dependent on STAT3 Tyr705 phosphorylation.

### TLR9 activation promoted STAT3 Tyr705 phosphorylation through PARP1-mediated STAT3 PARylation in HCC cells

**Figure 3:**
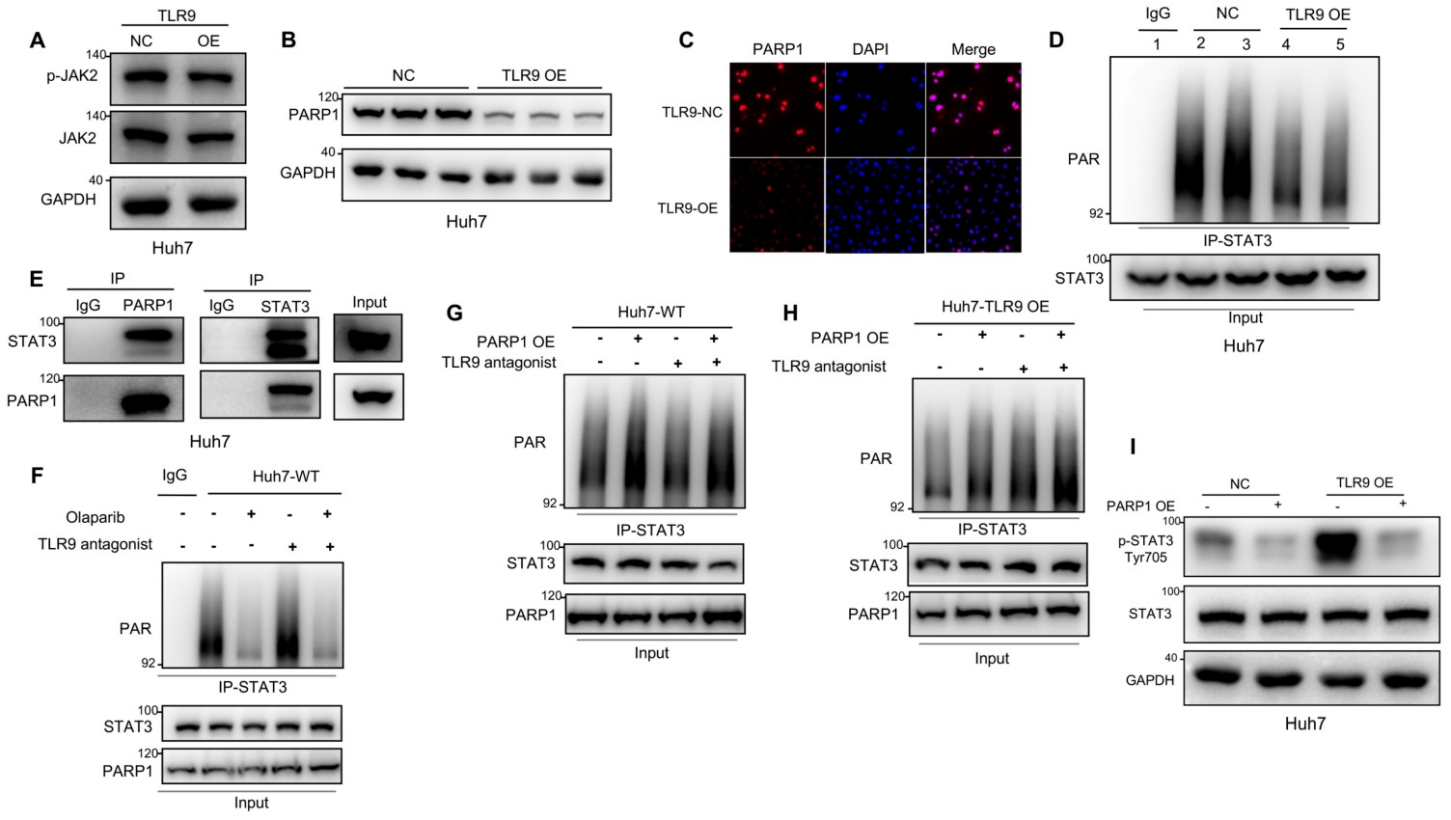
** TLR9 activation promoted STAT3 Tyr705 phosphorylation through PARP1-mediated STAT3 PARylation. (A)** p-JAK level after exogenous TLR9 overexpression in Huh7 cells. p-JAK expression levels were analyzed by Western blotting. (**B** and **C**) PARP1 levels after TLR9 overexpression. PARP1 levels were analyzed by Western blotting or immunofluorescence. (**D**) PARylated STAT3 level after TLR9 overexpression in Huh7 cells. Cell lysates were immunoprecipitated using anti-IgG or anti-STAT3 antibodies and immunoblotted using an anti-PAR antibody. (**E**) Interaction between endogenous STAT3 and PARP1. Huh7 cell lysates were immunoprecipitated with anti-PARP1 or anti-STAT3 antibodies, followed by immunoblotting with an anti-STAT3 or anti-PARP1 antibody. (**F**) PARylated STAT3 level in wild-type Huh7 cells in the presence of the PARP inhibitor olaparib or TLR9 antagonist chloroquine diphosphate. Cell lysates were immunoprecipitated using anti-IgG or anti-STAT3 antibodies and immunoblotted using an anti-PAR antibody. (**G** and **H**) PARylated STAT3 levels in wild-type (**G**) or TLR9-overexpressing (**H**) Huh7 cells in the presence of exogenous PARP1 overexpression or the TLR9 antagonist chloroquine diphosphate. Cell lysates were immunoprecipitated using anti-IgG or anti-STAT3 antibodies and immunoblotted using an anti-PAR antibody. (**I**) p-STAT3 (Tyr705) levels after exogenous PARP1 overexpression in TLR9-overexpressing Huh7 cells. p-STAT3 (Tyr705) levels were analyzed after TLR9 overexpression alone or in the presence of PARP1 overexpression.

STATs have been reported to be phosphorylated by JAK2 and to then dimerize and translocate to the nucleus, where they activate the transcription of other genes 44. Our data showed that JAK2 phosphorylation was not affected by TLR9 overexpression (Figure 3A), suggesting that the enhanced STAT3 Tyr705 phosphorylation induced by TLR9 activation may be associated with other signaling pathways. PARP1 can bind to and PARylate target proteins and thus affect protein function by synthesizing poly(ADP-ribose) (PAR) 45. STAT3 dephosphorylation has been reported to be regulated by PARP1-mediated STAT3 PARylation 39. To investigate the effects of TLR9 overexpression on PARP1 expression and STAT3 PARylation, we examined the protein levels of PARP1 and PAR pulled down by an anti-STAT3 antibody when TLR9 was overexpressed in Huh7 cells. PARP1 expression levels and PARylation of STAT3 were significantly decreased, indicating that TLR9 activation suppressed PARP1 expression and subsequently inhibited STAT3 PARylation (Figure 3B-D). Next, we investigated whether STAT3 PARylation regulated by TLR9 was dependent on PARP1 expression. We first used coimmunoprecipitation techniques to show that STAT3 can bind to PARP1 (Figure 3E). We next found that STAT3 PARylation was reduced when PARP1 was inhibited in wild-type Huh7 cells (Figure 3F). In contrast, STAT3 PARylation was restored after exogenous PARP1 upregulation in both wild-type and TLR9-overexpressing Huh7 cells (Figure 3G-H), suggesting that PARP1 PARylated STAT3 and regulated this modification. To investigate whether STAT3 PARylation affects STAT3 phosphorylation, we upregulated PARP1 expression in TLR9-overexpressing Huh7 cells. We found that the increased phosphorylation levels of STAT3 were significantly inhibited by the enhanced STAT3 PARylation induced by PARP1 (Figure 3I). These data indicate that TLR9 activation downregulates PARP1 levels and then inhibits STAT3 PARylation, ultimately increasing STAT3 phosphorylation.

### TLR9 activation promoted RNF146-induced PARP1 ubiquitin-mediated degradation through PARG-induced PARP1 autoPARylation

**Figure 4:**
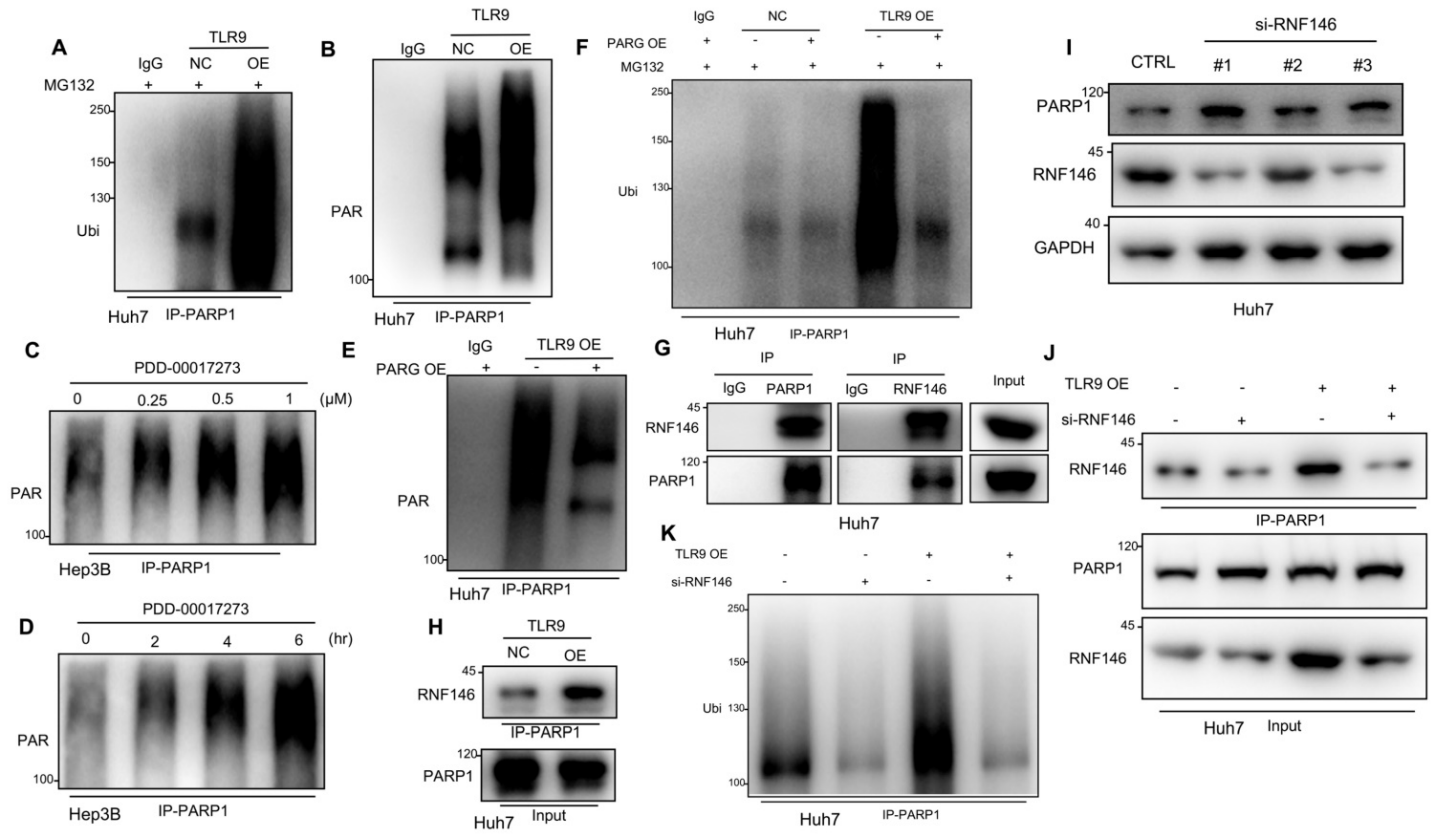
** TLR9 activation promoted RNF146-induced PARP1 ubiquitin-mediated degradation through PARG-induced PARP1 autoPARylation.** (**A**) Ubiquitination assay evaluating PARP1 after TLR9 overexpression in Huh7 cells. Cell lysates were immunoprecipitated with an anti-PARP1 antibody and subjected to Western blot analysis with an antibody against ubiquitin. The cells were treated with MG132 prior to the ubiquitination analysis. **(B)** AutoPARylation assay evaluating PARP1 after TLR9 overexpression in Huh7 cells. Cell lysates were immunoprecipitated with an anti-PARP1 antibody and subjected to Western blot analysis with an antibody against PAR. (**C** and **D**) AutoPARylation assay evaluating PARP1 in Hep3B cells after PARG inhibition with different concentrations (**C**) of inhibitor (PDD-00017273: 0, 0.25, 0.5, or 1 μM) or with 0.5 μM concentration (**D**) for the indicated times (times: 0, 2, 4, and 6 hours). Cell lysates were immunoprecipitated with an anti-PARP1 antibody and subjected to Western blot analysis with an antibody against PAR. (**E**) AutoPARylation assay evaluating PARP1 after TLR9 overexpression alone or with exogenous PARG overexpression. Cell lysates were immunoprecipitated with an anti-PARP1 antibody and subjected to Western blot analysis with an antibody against PAR. (**F**) Ubiquitination assay evaluating PARP1 after TLR9 overexpression alone or with exogenous PARG overexpression. Cell lysates were immunoprecipitated with an anti-PARP1 antibody and subjected to Western blot analysis with an antibody against ubiquitin. The cells were treated with MG132 prior to the ubiquitination analysis. (**G**) Interaction between endogenous RNF146 and PARP1. Huh7 cell lysates were immunoprecipitated with anti-PARP1 or anti-RNF-146 antibodies, followed by immunoblotting with an anti-RNF146 or anti-PARP1 antibody. (**H**) Interaction between endogenous RNF146 and PARP1 after TLR9 overexpression. Huh7 cell lysates were immunoprecipitated with an anti-PARP1 antibody, followed by immunoblotting with an anti-RNF146 antibody. (**I**) PARP1 expression after RNF146 silencing with siRNA in Huh7 cells. PARP1 expression levels were analyzed by Western blotting. (**J**) Interaction between endogenous RNF146 and PARP1 after TLR9 overexpression alone or with RNF146 silencing. Huh7 cell lysates were immunoprecipitated with an anti-PARP1 antibody, followed by immunoblotting with an anti-RNF146 antibody. (**K**) Ubiquitination assay evaluating PARP1 after TLR9 overexpression alone or in the presence of RNF146-specific siRNA. Cell lysates were immunoprecipitated with an anti-PARP1 antibody and subjected to Western blot analysis with an antibody against ubiquitin.

Next, we investigated the mechanisms by which TLR9 negatively regulates PARP1 expression in HCC cells. PARP1 mRNA expression was quantified by real-time PCR after TLR9 agonist stimulation or TLR9 overexpression. The results showed that PARP1 mRNA expression was not affected by TLR9 activation in HCC cells (Figure S3A-B). Then, we detected the ubiquitination of endogenous PARP1 by coimmunoprecipitation and found that PARP1 ubiquitination was significantly increased after TLR9 overexpression (Figure 4A). Thus, PARP1 ubiquitin-mediated degradation was significantly increased after TLR9 overexpression. These results imply that PARP1 inhibition by TLR9 may not occur at the transcriptional level but instead occur at the posttranslational level. A previous study reported that PARP1 could be phosphorylated by MAPKs to regulate PARP1 degradation. In particular, JNK1/2 has been shown to phosphorylate PARP1 and promote PARP1 ubiquitination and degradation 46. The MAPK pathway is also downstream of TLR9, which can be activated and regulated by TLR9 signaling. In this study, we found that PARP1 downregulation after TLR9 overexpression was not associated with MAPK pathway activation in Huh7 cells, which ruled out the possibility that the decreased level of PARP1 induced by TLR9 activation was dependent on MAPK pathway activation in Huh7 cells (Figure S3C-E).

A previous study demonstrated that autoPARylated PARP1 could be ubiquitinated and then degraded by an E3 ubiquitin ligase 47. To investigate whether the PARP1 ubiquitin-mediated degradation promoted by TLR9 was due to PARP1 autoPARylation, we detected PAR following immunoprecipitation with antibodies specific for PARP1. The results showed that PARP1 autoPARylation was significantly increased after TLR9 overexpression (Figure 4B), suggesting that TLR9 promoted the autoPARylation of PARP1. As a PAR hydrolase, PARG has been reported to degrade target proteins after PAR modification 47, and the levels of PAR-modified proteins increase when PARG is inhibited 48. Because PARG expression was decreased when TLR9 expression was enhanced (Figure S3F), we wondered whether PARG is involved in regulating PARP1 autoPARylation. Following PARP1 immunoprecipitation, immunoblotting with antibodies specific for PAR revealed that PARP1 autoPARylation was significantly increased after pharmacological inhibition of PARG with PDD-00017273 in Hep3B cells (Figure 4C-D), indicating that PARG inhibition increases PARP1 autoPARylation in HCC cells. Furthermore, we evaluated the effect of PARG overexpression on the level of PARP1 PARylation by performing immunoprecipitation with antibodies specific for PARP1. We found that restoring PARG expression decreased the enhanced PARP1 PARylation in TLR9-overexpressing Huh7 cells (Figure 4E), indicating that TLR9 may increase PARP1 autoPARylation by inhibiting PARG expression. Furthermore, we examined the change in PARP1 ubiquitination after exogenous PARG overexpression and found that PARP1 ubiquitination was significantly reduced when PARG expression was enhanced in TLR9-overexpressing Huh7 cells (Figure 4F), suggesting that PARG regulates PARP1 ubiquitin-mediated degradation by promoting PARP1 autoPARylation.

Recent studies have reported that RNF146 is a PAR-dependent E3 ubiquitin ligase that can ubiquitinate PARylated proteins, including PARP1, and promote their degradation 45, 47. We tested whether RNF146 interacts with PARP1 in HCC, and an endogenous complex containing RNF146 and PARP1 was detected (Figure 4G). Interestingly, the interaction between RNF146 and PARP1 was significantly enhanced in TLR9-overexpressing cells (Figure 4H), which was consistent with the above finding that PARP1 autoPARylation was significantly increased after TLR9 overexpression (Figure 4B). To test whether RNF146 can act as an E3 ubiquitin ligase for PARP1, we knocked out RNF146 with siRNA in HCC cells. We observed that knocking out RNF146 increased the protein level of PARP1 (Figure 4I) and reduced the interaction between RNF146 and PARP1 (Figure 4J). Furthermore, the level of ubiquitinated PARP1 was significantly decreased, along with a decrease in the RNF146 level, in TLR9-overexpressing cells (Figure 4K), suggesting that the E3 ligase RNF146 promotes the ubiquitination and degradation of PARP1. Taken together, the above data indicate that TLR9 promotes the ubiquitin-mediated degradation of PARP1 and is dependent on PARG-mediated PARP1 autoPARylation.

### Correlations among the expression of TLR9, PARP1, p-STAT3 (Tyr705) and PD-L1 in mouse and human tumor tissue samples

**Figure 5:**
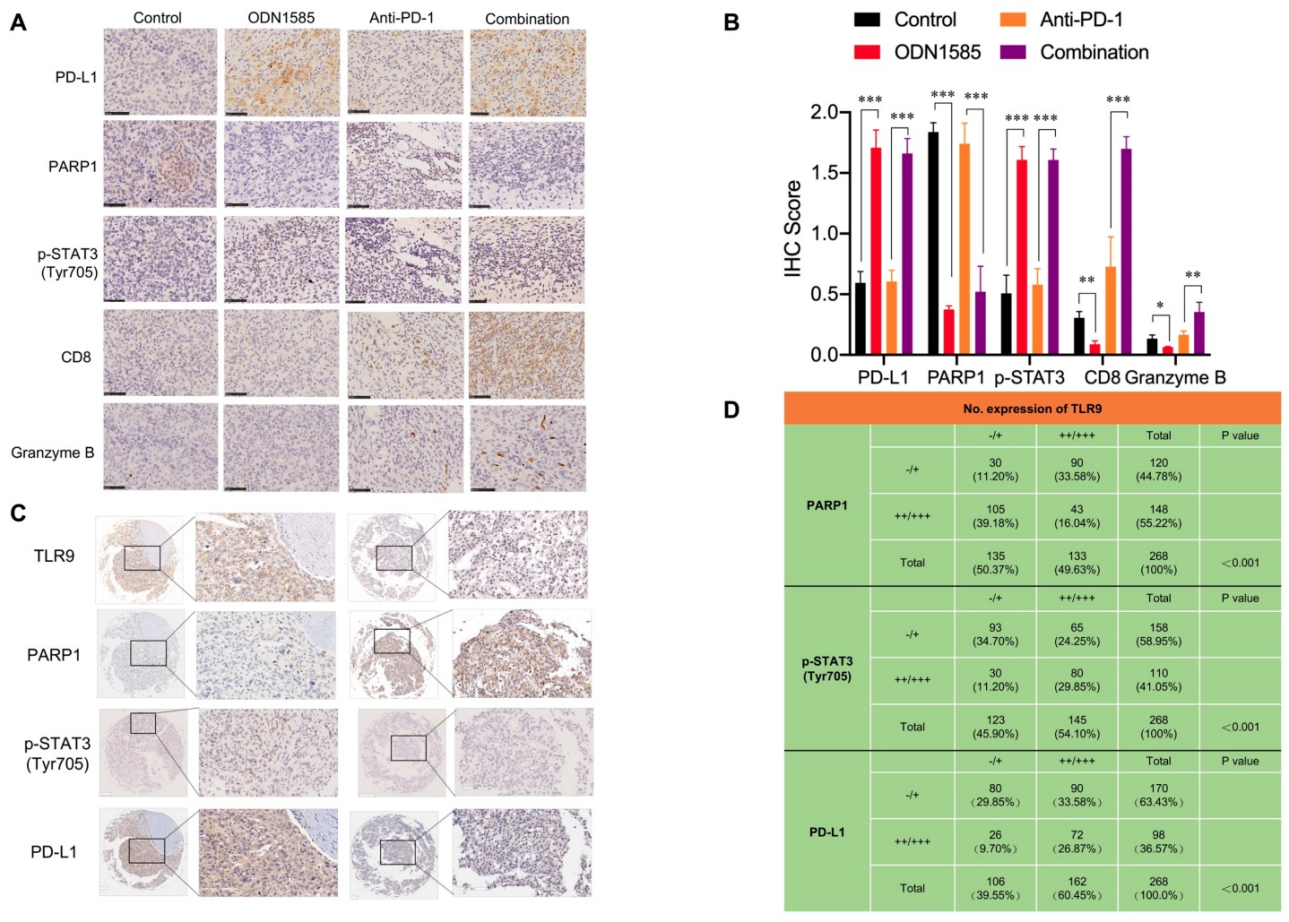
** Correlations among the expression of TLR9, PARP1, p-STAT3 (Tyr705) and PD-L1 in mouse and human tumor tissue samples.** (**A**) Immunohistochemical staining for PD-L1, PARP1, p-STAT3, CD8, and granzyme B protein expression patterns in Hepa1-6 tumors. Scale bar, 50 μm. (**B**) Histogram showing the immunohistochemistry score of PD-L1, PARP1, p-STAT3, CD8 and Granzyme B in each group. (values are mean ± SD, *p < 0.05, **p < 0.01, ***p<0.001). (**C**) Representative images of immunohistochemical staining of HCC tumors for TLR9, PARP1, p-STAT3 (Tyr705) and PD-L1. Patient tissue samples were stained for TLR9, PARP1, p-STAT3 and PD-L1. (**D**) Correlations between TLR9 levels and PARP1, p-STAT3 (Tyr705) or PD-L1 levels in liver cancer patients. p, Pearson chi-square test; -/+, negative or low expression; ++/+++, medium or high expression. Scale bar, 100 μm.

To further validate our findings in mouse and patient HCC samples, we analyzed the correlations among TLR9, PARP1, p-STAT3 and PD-L1 expression by performing immunohistochemical staining. The immunohistochemical analysis showed that the levels of PD-L1 and p-STAT3 (Tyr705) were increased, while that of PARP1 was decreased in tumor tissue samples from mice treated with ODN1585 alone or in combination with an anti-PD-1 antibody (Figure 5A-B, Figure S4A). To determine the effects of TLR9 activation on immune system, we used flow cytometry to analyze the immune cells from spleen and tumor after ODN1585 treatment. We observed a significant increase of DC cells CD4^+^ T cells and CD8^+^ T cells in spleen after TLR9 agonist treatment whereas the percentage of other immune cells, for example, regulatory T (Treg) cells, natural killer cells, monocyte and macrophage did not change significantly (Figure S4B). However, we found that the proportion of CD8^+^ T cells were significantly decreased despite of increase of DC and CD4^+^ T cells in tumor after TLR9 agonist stimulation (Figure S4C), suggesting that TLR9 agonist may induce immune escape by inhibiting T cell infiltration and activation in tumor microenvironment. In addition, we found that PD-1^+^ T cells such as, CD4^+^ PD-1^+^ T cells, CD8^+^ PD-1^+^ T cells, CD4^+^CD25^+^Foxp3^+^ PD-1^+^ T cells in mice spleen and liver tumor tissues did not change significantly after TLR9 agonist stimulation (Figure S4D-E), implying that TLR9 activation did not influence PD-1 levels in T cells. Moreover, the activated tumor-infiltrating CD8^+^ T cell population and granzyme B expression were increased in the mice treated with the ODN1585 and anti-PD-1 antibody combination (Figure 5B, Figure S4A), indicating that this combination treatment improved immune activity in the mice. These results suggested that ODN1585 might induce immunosuppression through upregulating PD-L1 expression and that the combination of ODN1585 and an anti-PD-1 antibody has potential therapeutic benefits.

As expected, in 268 human HCC tissue samples, the expression level of TLR9 was inversely correlated with that of PARP1 and positively correlated with that of p-STAT3 and PD-L1 (Figure 5C). Specifically, approximately 44.44% of the tumor samples with high TLR9 expression showed strong PD-L1 staining, and 75.47% of those with low TLR9 expression showed weak PD-L1 staining or no PD-L1 staining (p < 0.001) (Figure 5D). Similarly, approximately 55.17% of the tumor samples with high TLR9 expression showed strong p-STAT3 (Tyr705) staining, and 75.61% of those with low TLR9 expression showed weak p-STAT3 (Tyr705) staining (p < 0.001) (Figure 5D). In contrast, approximately 67.67% of the tumor samples with high TLR9 expression showed weak PARP1 staining or no staining, and 77.77% of those with low TLR9 expression showed strong PARP1 staining (p < 0.001) (Figure 5D). Taken together, these data indicate that high TLR9 expression is associated with low PARP1 expression and high p-STAT3 (Tyr705) and PD-L1 expression in mouse and patient HCC samples.

## Discussion

Clinical trial and animal research data have revealed that TLR9 agonists can warm “cold” melanoma tumors and reverse ICB resistance by expanding functional T cells 29, 30. The TLR signaling pathway is a double-edged sword in cancer therapy due to its immunostimulatory and immunosuppressive effects 18. It has been reported that stimulating tumor cells with a TLR9 agonist can lead to tumor cell death by inducing antitumor immunity associated with immunogenic or immunostimulatory cell death 15. However, the induction of TLRs, especially TLR9, in tumor cells by endogenous ligands not only fails to result in antitumor immunity but rather contributes to tumor progression 18, 23, 24, 27, which may contribute to the failure of TLR9 agonists in systemic antitumor therapy in clinical trials 16, 17. A critical question is why a drug that may induce immunosuppression can enhance the antitumor effect of ICB. In this paper, we addressed this critical issue using a clinically relevant mouse model of HCC. First, we found that a TLR9 agonist in combination with anti-PD-1 therapy or anti-PD-L1 therapy boosted antitumor efficacy in an HCC mouse model (Figure 1B-E and Figure S1F-I). Moreover, the TLR9 agonist ODN1585 failed to significantly reduce the tumor burden and led to T cell decrease in the treated group compared with the control group (Figure 1B, Figure 5A-B, Figure S4A and C), which was consistent with the findings of clinical trials 16, 17. Second, we found that TLR9 activation upregulated PD-L1 expression in HCC cells, which led to the suppression of antitumor immunity. Importantly, this finding may provide strong evidence for combining a TLR9 agonist and an anti-PD-1 antibody in antitumor therapy because ICB can overcome the immunosuppression induced by TLR9 activation, while TLR9 agonists can enhance anti-PD-1 therapy response rates for its upregulation of PD-L1 expression.

Regarding the mechanism, we found TLR9 signaling and STAT3 activation in tumor cells. Upon TLR9 activation, STAT3 phosphorylation at Tyr705 was increased, which led to PD-L1 expression upregulation in HCC cells (Figure 2K-L), suggesting that tumor cells can be directly affected by TLR9 ligation to escape immune attack. STAT3 is constitutively activated by Tyr705 and Ser727 phosphorylation in diverse cancers of either hematopoietic or epithelial origin, and STAT3 prevents apoptosis and enhances tumor cell proliferation and survival after activation 49-51. Emerging oligonucleotide-based strategies to inhibit STAT3 signaling, such as STAT3-siRNA linked to a CpG oligonucleotide agonist of TLR9 that can target and silence STAT3 in tumor-related immune cells in the tumor microenvironment, have shown two-pronged activating effects as antitumor therapies 40, 52-54. Phosphorylated STAT3 can dimerize and translocate to the nucleus, where it acts directly on the promoter of PD-L1 to increase PD-L1 expression in human cancer cells. The activity of STAT3 is negatively regulated by tyrosine phosphatases when phosphorylation is inhibited. Despite its critical role in tumor cell proliferation and survival, the posttranscriptional fate of STAT3 has not been thoroughly defined. Ding *et al* revealed that the transcriptional activity of STAT3 was inhibited when STAT3 was PARylated by PARP1, showing that PARP1 could be a suppressor of STAT3 phosphorylation in cancer cells 39. In this study, we unexpectedly found that PARP1 inhibition by TLR9 (Figure 3B-C) led to a decrease in the PARylation and an increase in the Tyr705 phosphorylation of STAT3 to increase PD-L1 expression (Figure 3D, Figure 3I), which indicated that TLR9 regulated the crosstalk of STAT3 PARylation and phosphorylation by affecting PARP1 expression in HCC cells.

PARP1, the most characterized member of the PARPs, is an abundant nuclear protein with enzymatic activity that can be activated in response to DNA damage and promotes the formation of a poly(ADP-ribose) polymer (pADPr) on its substrates as well as itself, which then regulates the modulation of protein stabilization, as well as protein-protein interaction scaffold localization and formation 45. Hu *et al* revealed that PARP1 bound to and PARylated BRD7 and induced the ubiquitin-mediated degradation of BRD7 through a PAR-binding E3 ubiquitin ligase, which leads to cancer cell resistance to DNA-damaging agents 45. Li *et al.* reported that PARP1 interacted with HMGB1 and accelerated its translocation from the nucleus to the cytoplasm, which ultimately led to cardiac hypertrophy 55. High PARP1 expression was found to lead to acquired chemotherapeutic drug resistance, and PARP1 has thus been explored for its therapeutic potential in cancer treatment 56. However, the mechanism of PARP1 protein level regulation remains poorly understood. Wang *et al* demonstrated that the PARP1 level was maintained by MKP-1-dependent JNK inactivation, which inhibited PARP1 ubiquitin-mediated degradation 46. Singo *et al* demonstrated a PARP1 regulatory mechanism, in which Nutlin could induce the proteasomal degradation of PARP1 in a p53-dependent manner 57. In this study, we identified a novel mechanism in which TLR9 negatively regulated PARP1 by promoting PARG-induced PARP1 autoPARylation, which led to the RNF146-dependent ubiquitin-mediated degradation of PARP1. PARG, an important enzyme that hydrolyzes PAR on PARylated target proteins, was found to be inhibited when TLR9 was overexpressed in HCC (Figure S3F). This inhibition of PARG then led to an increase in the autoPARylation of PARP1 (Figure 4C-D), which was consistent with the loss of PARG restoring the PAR formation on PARP1 that conferred resistance to PARP inhibition in BRCA2-deficient tumor cells 48. This novel regulatory mechanism involving TLR9 in the posttranscriptional modification of PARP1 and the level of PARG may contribute to the development of a cancer therapy involving the PARP or PARG signaling pathway.

## Conclusions

**Figure 6:**
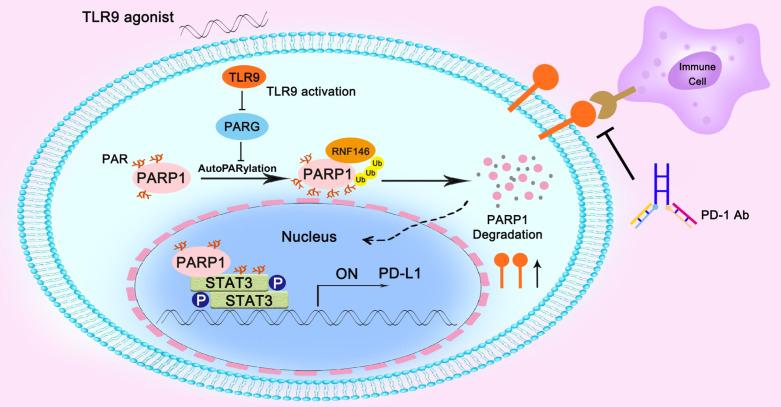
** Illustration of the proposed working model.** TLR9 regulated the crosstalk between PARP1 autoPARylation and ubiquitination and between STAT3 PARylation and phosphorylation, which together participate in PD-L1 transcription expression.

In summary, we found that the combination of a TLR9 agonist and an anti-PD-1 monoclonal antibody could boost the efficacy of antitumor immunity. Mechanistically, we found that inhibiting PARG expression by TLR9 led to enhanced PARP1 autoPARylation, thereby increasing RNF146-mediated PARP1 ubiquitin-mediated degradation, which resulted in a significant reduction in PARP1 expression levels. Then, the suppressed PARP1 expression resulted in decreased STAT3 PARylation and increased STAT3 phosphorylation at Tyr705, ultimately promoting PD-L1 transcription, which led to the suppression of antitumor immunity (Figure 6).

## Supplementary Material

Supplementary figures and tables.

## Abbreviations

HCC: Hepatocellular carcinoma
ICB: Immune checkpoint blockade
TLRs: Toll-like receptors
TLR9: Toll-like receptors 9
PARP1: Poly ADP-ribose polymerase 1
STAT: Signal transducer and activator of transcription
PD-L1: Programmed cell death ligand 1
PD-1: Programmed cell death 1
PCR: Polymerase chain reaction
RNF-146: RING finger protein 146
PARG: Poly (ADP-ribose) glycohydrolase
IRF1: Interferon regulatory factor-1
IRF3: Interferon regulatory factor-3
MAPK: Mitogen-activated protein kinases
siRNA: Small interfering ribonucleic acid
