# Supplementary material for: Hepatoma cell-intrinsic TLR9 activation induces immune escape through PD-L1 upregulation in hepatocellular carcinoma
Source: Theranostics. 2020 May 17;10(14):6530–43. doi: 10.7150/thno.44417 (PMC7255037; doi:10.7150/thno.44417)
Supplement: Supplementary file 1 — Supplementary figures and tables. [file thnov10p6530s1.pdf]

Supplementary Information

The contents in this section include four supplementary figures.

Supplementary Figure. 1

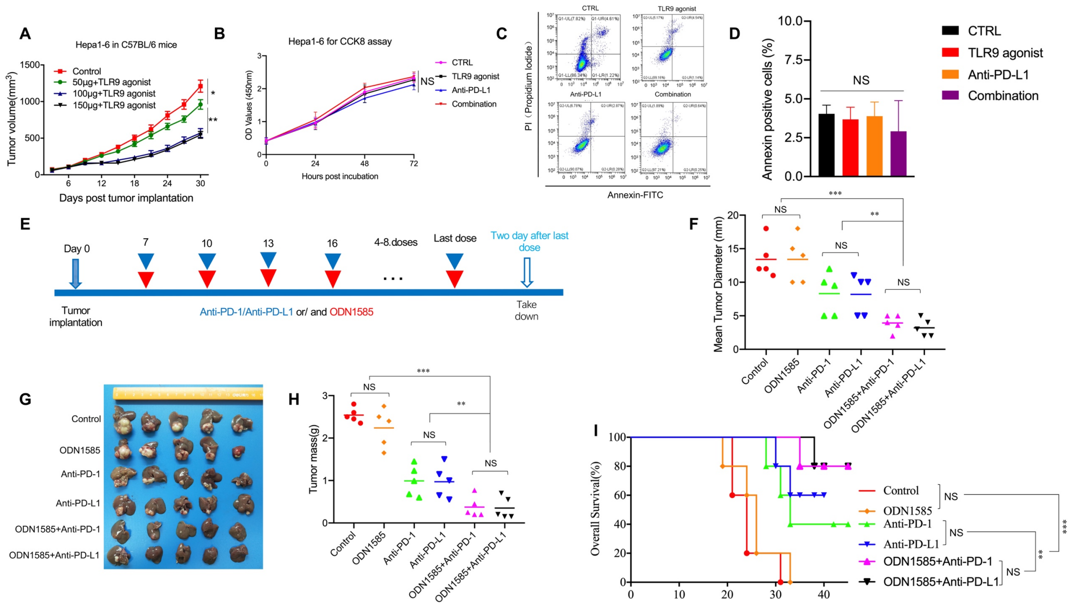

(**A**) Growth of subcutaneous Hepa1-6 tumors in ODN1585 combine with anti-PD-1 antibody or control-treated C57BL/6 mice. Tumors were measured at the indicated time points. (*n*=5 per group, values are mean ± SD, *p <0.05, **p<0.01). (**B**) Untreated Hepa1-6 cells or cells treated with ODN1585 (murine TLR9 agonist, 5μM) or anti-PD-L1 (0.5μg/μl) or combination for 48 h were subjected to a Cell Counting Kit-8 assay. The viable cells were measured after 24, 48, and 72 h. (values are mean ± SD, NS indicates no significance). (**C** and **D**) Flow cytometry analysis of apoptotic cells under after treatment with TLR9 agonist or anti-PD-L1. (values are mean ± SD, NS indicates no significance). (**E**) Schematic diagram of the drug intervention protocol utilizing the TLR9 agonist ODN1585 and/or anti-PD-1 antibody or anti-PD-L1 antibody to treat C57BL/6 mice. (**F**) Mean tumor diameter of orthotopic Hepa1-6 tumors in ODN1585 and/or anti-PD-1 antibody or anti-PD-L1 antibody treated C57BL/6 mice. (*n*=5 per group, **p < 0.01, ***p<0.001, NS indicates no significance). (**G**) Representative images of Hepa1-6 orthotopic HCC tumors from each group. (**H**) Tumor weights at the drug intervention endpoints. (*n*=5 per group, **p < 0.01, ***p<0.001, NS indicates no significance). (**I**) Survival of mice bearing Hepa1-6 tumors following treatment with ODN1585 and/or anti-PD-1 antibody or anti-PD-L1 antibody. (*n*=5 per group, **p < 0.01, ***p<0.001, NS indicates no significance).

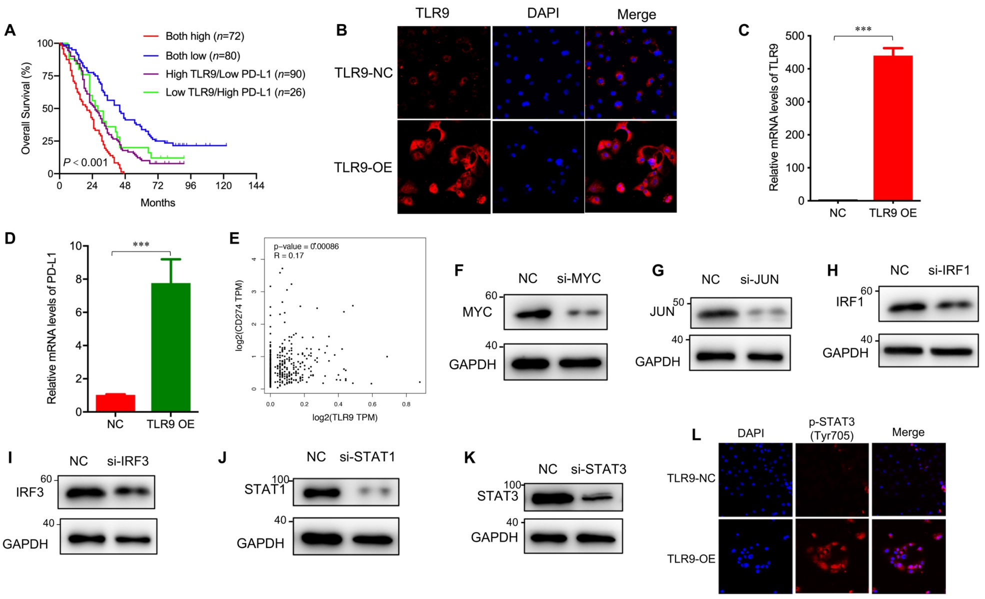

Supplementary Figure. 2

(**A**) The overall survival of difference groups according to the expression pattern of TLR9 and PD-L1 were analyzed with Kaplan-Meier survival analysis. (**B**) TLR9 expression after exogenous TLR9 overexpression in Huh7 cells. The TLR9 expression levels were analyzed by immunofluorescence. (**C**) mRNA levels of TLR9 measured by qRT-PCR after exogenous TLR9 overexpression in Huh7 cells. (values are mean ± SD, ***p<0.001). (**D**) mRNA levels of PD-L1 measured by qRT-PCR after exogenous TLR9 overexpression in Huh7 cells. (values are mean ± SD, ***p<0.001). (**E**) Correlation of TLR9 expression to PD-L1 expression by using the Signature Score Function at the GEPIA server. (**F-K**) MYC, JUN, IRF1, IRF3, STAT1 and STAT3 expression after silencing with siRNA in Huh7 cells. The MYC (**F**), JUN (**G**), IRF1 (**H**), IRF3 (**I**), STAT1 (**J**) and STAT3 (**K**) expression levels were analyzed by Western blotting. (**L**) p-STAT3 (Tyr705) levels after exogenous TLR9 overexpression in Huh7 cells. p-STAT3 levels were analyzed by immunofluorescence.

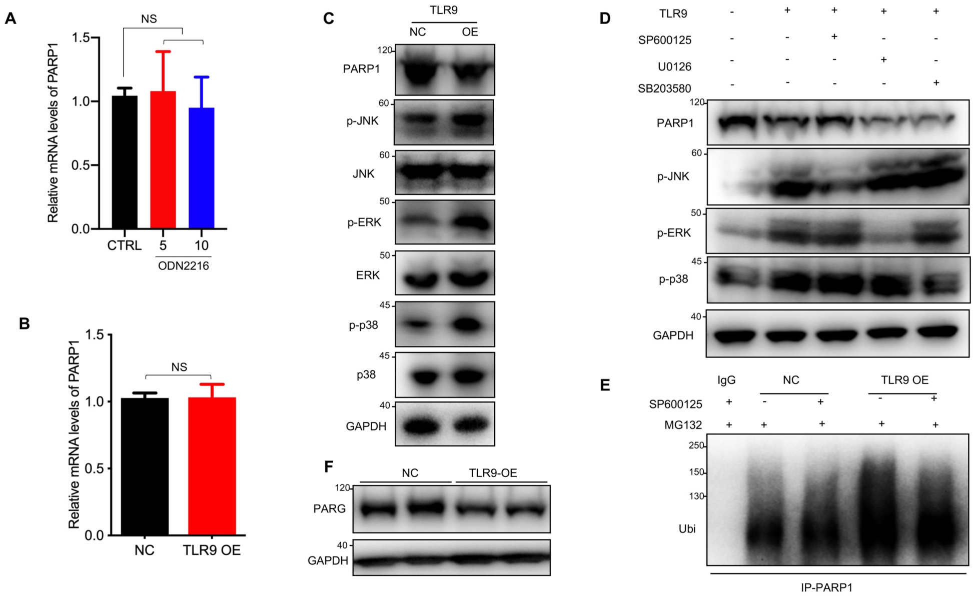

Supplementary Figure. 3

(**A**) mRNA levels of PARP1 in Hep3B cells measured by qRT-PCR after exogenous TLR9 agonist stimulation. (values are mean ± SD, NS indicates no significance). (**B**) mRNA levels of PARP1 in Huh7 cells measured by qRT-PCR after exogenous TLR9 overexpression. (values are mean ± SD, NS indicates no significance). (**C**) PARP1 and MAPK levels after TLR9 overexpression. PARP1, p-JNK, p-ERK, and p-p38 levels were analyzed by Western blotting. (**D**) PARP1 levels after TLR9 overexpression alone or in the presence of the MAPK inhibitors SP600125, U0126, and SB203580. The PARP1, p-JNK, p-ERK, and p-p38 levels were analyzed by Western blotting. (**E**) Ubiquitination assay evaluating PARP1 after TLR9 overexpression alone or in the presence of the JNK inhibitor SP600125. Cell lysates were immunoprecipitated with an anti-PARP1 antibody and subjected to Western blot analysis with an antibody against ubiquitin. The cells were treated with MG132 prior to the ubiquitination analysis. (**F**) PARG levels after TLR9 overexpression. PARG levels were analyzed by Western blotting.

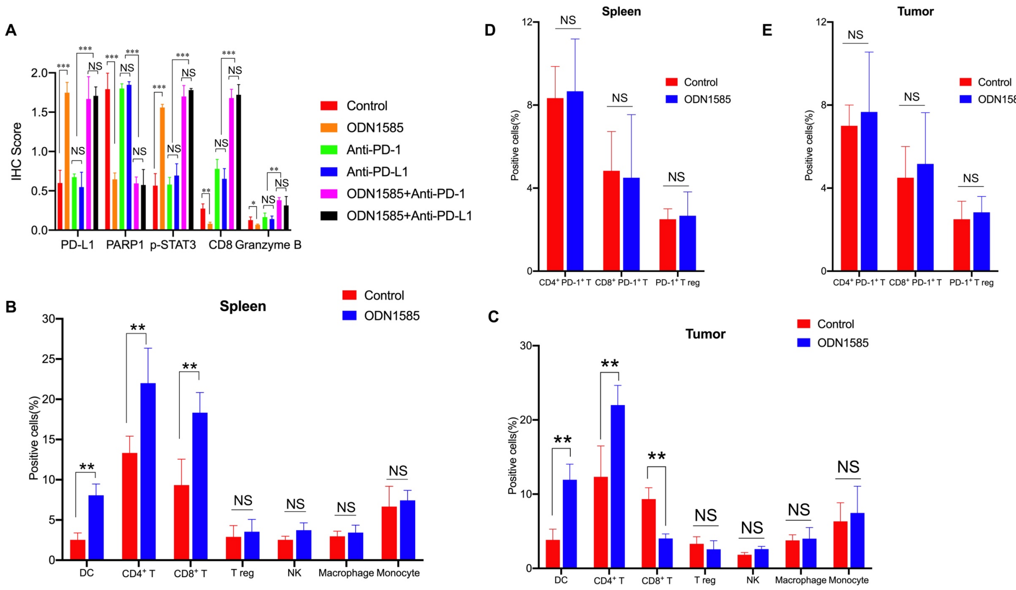

Supplementary Figure. 4

(**A**) Histogram showing the immunohistochemistry score of PD-L1, PARP1, p-STAT3, CD8 and Granzyme B in Hepa1-6 tumors following treatment with TLR9 agonist ODN1585 and/or anti-PD-1 antibody or anti-PD-L1 antibody. (values are mean ± SD, *P < 0.05, **p < 0.01, ***p<0.001, NS indicates no significance). (**B** and **C**) Immune cells, including T cells ($CD4^+$ and $CD8^+$), T regulatory (Treg) cells ($CD4^+CD25^+Foxp3^+$), natural killer (NK) cells ($NK1.1^+$), macrophage cells ($F4/80^+CD11b^+$), monocyte cells ($CD11b^+$) and dendritic cells ($CD11c^+CD317^+$) were detected by flow cytometry in mice spleen (**B**) and liver tumor tissues (**C**). (values are mean ± SD, **p < 0.01, NS indicates no significance). (**D** and **E**) $PD\text{-}1^+$ T cells, including $CD4^+PD\text{-}1^+$T cells, $CD8^+PD\text{-}1^+$T cells, $CD4^+CD25^+Foxp3^+PD\text{-}1^+$ T cells were detected by flow cytometry after ODN1585 treatment in mice spleen (**D**) and liver tumor tissues (**E**). (values are mean ± SD, NS indicates no significance).
